# The Accuracy of Pre-Endoscopic Scores for Mortality Prediction in Patients with Upper GI Bleeding and No Endoscopy Performed

**DOI:** 10.3390/diagnostics13061188

**Published:** 2023-03-21

**Authors:** Sergiu Marian Cazacu, Dragoș Ovidiu Alexandru, Răzvan-Cristian Statie, Sevastița Iordache, Bogdan Silviu Ungureanu, Vlad Florin Iovănescu, Petrică Popa, Victor Mihai Sacerdoțianu, Carmen Daniela Neagoe, Mirela Marinela Florescu

**Affiliations:** 1 Research Center of Gastroenterology and Hepatology, Gastroenterology Department, University of Medicine and Pharmacy Craiova, Petru Rares Street No 2-4, 200349 Craiova, Dolj County, Romania; 2 Biostatistics Department, University of Medicine and Pharmacy Craiova, Petru Rares Street No 2-4, 200349 Craiova, Dolj County, Romania; 3 Clinical Emergency County Hospital Craiova, 200642 Craiova, Dolj County, Romania; 4 Pathology Department, University of Medicine and Pharmacy Craiova, Petru Rares Street No 2-4, 200349 Craiova, Dolj County, Romania

**Keywords:** upper gastrointestinal bleeding, endoscopy, Glasgow-Blatchford score, Rockall score, AIM65, Iino score, MELD, Child–Pugh–Turcotte score

## Abstract

(1) Background: The assessment of mortality and rebleeding rate in upper gastrointestinal bleeding (UGIB) is essential, and several prognostic scores have been proposed. Some patients with UGIB did not undergo endoscopy, either because they refused the procedure, suffered from alcohol withdrawal symptoms or altered general status, or because the bleeding was severe enough to cause death before the endoscopy. The mortality risk in the subgroup of patients without endoscopy is poorly evaluated in the literature. (2) Methods: The purpose of the study was to identify the most useful scores for the assessment of in-hospital mortality in patients with UGIB with no endoscopy performed and no known etiology. A total of 198 patients with UGIB and no endoscopy performed were admitted between January 2017 and December 2021 and the accuracy of 12 prognostic scores and the Charlson comorbidity index for in-hospital mortality prediction were analyzed, as well as Child–Pugh Turcotte (CPT) and Meld scores in patients with cirrhosis. (3) Results: The mortality rate was 37.9%, higher than in variceal (21.9%, *p* < 0.0001) and non-variceal bleeding (7.4%, *p* < 0.0001). The most accurate scores by AUC were the International Bleeding score (INBS, 0.844), Glasgow Blatchford (0.783), MAP score (0.78), Iino (0.766), AIM65 and modified N-score (0.745 each), modified Glasgow-Blatchford (0.73), H3B2 and N-score (0.701); Rockall, Baylor, and T-score had an AUC below 0.7. MELD score was superior to CPT in patients with cirrhosis (AUC 0.811 versus 0.670). (4) Conclusions: The mortality rate in UGIB with no endoscopy was higher than in both variceal and non-variceal bleeding and was higher in the pandemic period but with no statistical significance (45.3% versus 32.14%, *p* = 0.0586), mainly because of positive cases. Only one case of rebleeding was noted; the hospitalization period was significantly shorter. The most accurate score was International Bleeding Score; the MELD score had a higher but moderate accuracy compared with CPT in patients with cirrhosis.

## 1. Introduction

Upper gastrointestinal bleeding (UGIB) represents a common cause of hospital admissions in the gastroenterology and intensive care unit (ICU), with mortality ranging from 3 to 15% [1,2,3,4]. In most cases, early treatment and early endoscopy during the first 24 h is recommended according to current guidelines. Risk assessment is also recommended, according to most current guidelines, in order to appropriately time interventions, including endoscopy, blood requirement, rebleeding risk, level of care, and also the risk of death [2].

Despite advances in endoscopic techniques, the mortality rate is still high in patients with variceal and non-variceal upper gastrointestinal bleeding (NVUGIB). The assessment of prognosis and rebleeding rate therefore continues to be a serious issue. A significant number of prognostic scores were proposed in order to evaluate the risk of mortality, the need for intervention and transfusions, the risk for rebleeding and ICU admission, and the length of admission [5]. Most scores were focused on clinical and/or endoscopic parameters and were perfectible. The first scores proposed were the Glasgow-Blatchford score (GBS) and Rockall (pre-endoscopic = RS pre and full = RS full) score; GBS was originally introduced as an instrument for assessing the need for intervention (blood transfusion, therapeutic endoscopy or surgery) and a modified Glasgow-Blatchford score to eliminate melena, syncope, and comorbidities [6,7], while RS was originally suggested as a tool for mortality prediction [3,5]. Other prognostic scores were Baylor bleeding score (BBS), Cedars–Sinai Medical Center Predictive Index (CSMCPI), AIM65 (which incorporates albumin level, hemoglobin, altered level of consciousness, INR, age and systolic blood pressure), and T-score (TSC). Baylor bleeding score (BBS) was initially developed in order to predict rebleeding risk, but performed poorly for mortality prediction in one study [1]. GBS and RS are complex and sometimes difficult to use in emergency settings due to their low accuracy. Thus, during the last 10 years, other scores have been proposed for predicting intervention, including the N-score [8], Iino score [7,9], and H3B2 [7], or for predicting mortality, such as the MAP score [10,11], INBS (International Bleeding Score), PNED (Progetto Nazionale Emmoragia Digestive) [12], and Harbinger score [13]. H3B2 was proposed in 2019 and is useful for the prediction of therapeutic interventions, including emergency endoscopy, with an accuracy superior to both GBS and modified GBS, and also with satisfactory accuracy in mortality prediction [7]. Iino or Hirosaki score was proposed in 2016 for predicting intervention in a study of 212 patients in Japan and demonstrated superiority to both clinical Rockall and AIM65 [14]. MAP score was proposed in 2020 for the prediction of both intervention and risk of death [10], and INBS (ABC score) was proposed in an international cohort study in order to better assess mortality risk [15] and subsequently validated in other studies as superior to preclinical Rockall, GBS, PNED and AIM65 [16,17].

Most studies regarding risk scores were evaluated in studies including all UGIB or non-variceal UGIB. Prognosis in variceal bleeding is related especially to the severity of liver failure (assessed using Child–Pugh–Turcotte or MELD scores); most studies on variceal bleeding focusing on the importance of classical prognostic scores concluded that the value was inferior in variceal bleeding compared to non-variceal bleeding [18,19,20]; a meta-analysis of 28 studies found that the Child–Pugh–Turcotte (CPT) score has better accuracy than classical prognostic scores (AIM65, GBS and Rockall), with moderate accuracy for all abovementioned scores [21], and some studies found that MELD score was better than CPT for predicting mortality [22]. The main limitation of prognostic scores in variceal bleeding may be the fact that only a few of them (AIM65, ABC) are correlated to the severity of the liver failure. Some improved CPT variants including creatinine level were also proposed in order to increase accuracy [23].

Although early endoscopy is recommended in all patients with UGIB, there are some patients who did not undergo endoscopy at all. Some of them refused the procedure, while in other cases, the general status was so altered (especially cardio-respiratory failure) that the procedure was postponed, and in some cases, not performed at all. In rare cases, the bleeding was so severe that the patients died before an endoscopy was performed; some cases of variceal bleeding were stopped because of Sengstaken–Blakemore tube placement, but the patients died due to liver failure. In some cases, alcohol withdrawal symptoms were so severe that endoscopy was not performed during admission. Therefore, the patients with no endoscopy performed represent a heterogenous group, with few studies regarding both mortality and the role of risk (non-endoscopic) scores in the prognostic evaluation. The mortality risk in the subgroup of patients without endoscopy is poorly evaluated in the literature; most studies included only patients with UGIB with endoscopy performed, and the absence of endoscopy represents one of the exclusion criteria in studies evaluating the performance of risk scores [1,3,4,10,24,25,26,27]. In this setting, a study evaluating the accuracy of prognostic scores for mortality in a subgroup of patients without endoscopy and with no known etiology for bleeding can be useful in order to predict the risk of death.

## 2. Materials and Methods

We retrospectively included in our study all patients who presented with UGIB in the emergency department and were admitted into the hospital during 2017–2021 (5 years). Diagnosis of UGIB was made by the presence of hematemesis (red or coffee-ground emesis), melena, or hematochezia and confirmed by upper digestive endoscopy; in patients with no endoscopy performed and with melena or hematochezia,, bleeding in the upper digestive tract was confirmed using a nasogastric tube placement. The study was conducted in accordance with the Declaration of Helsinki. Informed consent was obtained from all admitted patients and approval by the Local Ethics Committee of the Emergency Clinical County Hospital of Craiova was also obtained. Patients aged below 16 years and those who denied consent for data usage were excluded from the study.

### 2.1. Aim of the Study

The purpose of the study was to assess mortality in patients with no endoscopy performed compared with those with variceal and non-variceal bleeding to identify the most useful score/parameters for the assessment of in-hospital mortality and rebleeding rate, and to evaluate the influence of COVID-19 infection and pandemic period to endoscopy rate and mortality in patients with no endoscopy performed.

### 2.2. Data Collection

Parameters included in the study were: clinical (age, gender, syncope, altered level of consciousness, melena, hematemesis with blood or “ground coffee” hematemesis, blood pressure and heart rate, blood transfusion, use of NSAID, anticoagulants or antiplatelet agents, alcohol consumption, presence of comorbidities), biological (hemoglobin level, platelet count, INR, urea, albumin and creatinine level) and endoscopy (if performed). The evaluation of patients for COVID-19 infection during the pandemic period (March 2020–December 2021) was based on epidemiologic triage and, in case of suspicion of infection, pulmonary X-ray and PCR testing were performed; after 1 January 2021, rapid antigen testing was used for triage in all patients before admission. Most patients were examined by endoscopy in the first 24 h; patients with COVID-19 infection were managed conservatively, if possible, except for cases with hemodynamic instability or those with ongoing bleeding, when evaluation by emergency endoscopy in specially designated endoscopy rooms was implemented.

Four categories of patients with UGIB were selected: variceal UGIB (VUGIB), non-variceal UGIB (NVUGIB), obscure bleeding (when endoscopy was performed but no bleeding source was found and no anticoagulant over-dosage was noted) and UGIB of unknown origin and no endoscopy (patients with hematemesis or melena and bloody or coffee ground aspirate on nasogastric tube but with no endoscopy performed).

Clinical, biological, and endoscopic parameters were included in the database, and prognosis scores were calculated for every patient (Table 1, Table 2, Table 3, Table 4, Table 5, Table 6, Table 7, Table 8, Table 9 and Table 10). We evaluated classic scores (GBS and modified GBS, RS score, AIM65, T score, BBS), new scores (N-score, H3B2 score, Iino score, MAP score and INBS) and also the Charlson comorbidity index (CCI). Because bloody hematemesis was generally considered to signify a more severe bleeding, we tried to propose a modified N-score (m-N-score), which allocated 1 point for ground-coffee hematemesis and 2 points for red blood hematemesis (Table 6).

### 2.3. Statistical Analysis

The main outcome followed was in-hospital mortality; other outcomes were the need for transfusion and mean hospital days. Statistical data were analyzed and provided using IBM SPSS 29.0.0.0. Continuous variables were compared using the Mann–Whitney test if they were not found to have a normal (Gaussian) distribution after a test distribution with the Kolmogorov–Smirnov normality test, while for categorical variables, the Chi-square test or Fisher were used. For the accuracy prediction of prognostic scores, the AUC (Area operator Under Curve) was constructed, and cut-off values were established for prognostic scores.

## 3. Results

### 3.1. Patient Characteristics

A total of 2455 patients with UGIB were selected during the analyzed period. A total of 1617 were males and 838 were females. In total, 66.3% were non-variceal bleeding and 21.2% were variceal bleeding. In 198 cases (8.1%), the etiology was unknown because endoscopy was not performed; in 109, upper digestive endoscopy was followed by colonoscopy and in 8 cases, capsule endoscopy or spiral endoscopy could not establish the source of bleeding (obscure bleeding). The characteristics of patients with variceal, non-variceal, and unknown etiology bleeding are described in Table 11.

The mortality rate in patients with no endoscopy was much higher than in both variceal bleeding (OR = 2.1716, 95% CI 1.5235 to 3.0953, *p* < 0.0001) and non-variceal bleeding (OR = 7.5942, 95% CI 5.3962 to 10.6875, *p* < 0.0001). In patients with no endoscopy, during the pre-pandemic period, 36 deaths from 112 patients were recorded (mortality rate 32.1%), and during the pandemic period, 39 deaths were noted (18 patients tested positive for COVID-19, 11 tested negative, and 10 were not tested), while 47 survived (5 tested positive, 25 tested negative, and 17 not tested). The mortality was higher in the pandemic period compared with the pre-pandemic period but with no statistical significance (45.3% versus 32.14%, OR = 1.7518, 95% CI 0.9799 to 3.1317, *p* = 0.0586) and was higher in positive cases (62.1% versus 16.7%, OR = 8.1818, 95% CI 2.4192 to 27.6707, *p* = 0.0007). By removing positive cases from the pandemic period, the mortality was similar in both pre-pandemic and pandemic periods (33.3% versus 32.1%, OR= 1.0556, *p* = 0.8719).

Age was similar in patients with no endoscopy compared with non-variceal bleeding (65 versus 63.9 years, Chi-square test *p*-value 0.875658) and higher than in variceal bleeding (65 versus 58.7, Chi-square test *p*-value < 0.00001); similar male percentage was noted in cases of bleeding with no endoscopy in comparison with non-variceal bleeding (60.1% males in patients with no endoscopy, 67.6% in non-variceal bleeding-*p* value 0.0341, and 65.8% in variceal bleeding-*p* value 0.1573).

Cardiovascular and renal comorbidities in patients with no endoscopy were similar to NVUGIB (12.6% versus 14.9%, *p*-value = 0.4005, and 7.1% versus 7.7%, *p*-value= 0.7610, respectively), but higher proportions of both cirrhosis and metastatic malignancy were noted (35.9% versus 11% and 4.6% versus 0.4%, *p*-value < 0.0001 in both cases). Higher proportions of cirrhosis can be explained by a proportion of variceal bleeding in cases with unknown etiology; in 32 of 71 cases of cirrhosis, Sengstaken–Blakemore tube placement was performed, while in the remaining cases, the bleeding stopped before tube placement, patients did not consent to the placement, or the placement was not successful.

A lower percentage of use of anticoagulants (4% versus 13.3% *p*-value = 0.0004) and NSAID (4% versus 15.4%, *p*-value = 0.0001) were noted in patients with no endoscopy; for antiplatelet agents, the difference was not statistically significant (3% versus 6.5%, *p*-value = 0.0603).

Similar mean levels of hemoglobin (8.39) were noted in patients with no endoscopy compared with both NVUGIB (8.77 g/dL, *p* = 0.0768) and VUGIB (8.28 g/dL, *p* = 0.0768); blood transfusion was noted in 34.8% of patients with no endoscopy compared with 45.9% in non-variceal bleeding (*p* = 0.0032), and 55.2% in variceal bleeding (*p* < 0.0001). The mean value of serum albumin was lower compared with non-variceal bleeding (2.82 versus 3.27 g/dL, *p* < 0.0001), which may reflect a higher proportion of cirrhosis; mean creatinine level was higher compared with both non-variceal (1.56 mg/dL versus 1.24 mg/dL, *p* = 0.0060) and variceal bleeding (0.96 mg/dL, *p* < 0.0001); urea mean value was similar compared with non-variceal bleeding (90.7 versus 82.6 mg/dL, *p* = 0.0984), but higher compared with variceal bleeding (65.1, *p* < 0.0001), while INR mean values were similar (1.77 versus 1.58, *p* = 0.1551 and 1.77 versus 1.74, *p* = NS).

Mean onset to admission time (37.1 h) was intermediate between variceal bleeding (24.4 h, *p* = 0.0016) and non-variceal bleeding (46.7 h, *p* = 0.1194), which may reflect the fact that the unknown bleeding group actually represents a mixed population of both variceal and non-variceal cases.

Only one rebleeding case was noted, which was resolved with conservative treatment. The hospitalization period was significantly shorter (with more than 2 days) compared with both variceal and non-variceal cases (*p* < 0.0001 and 0.0002, respectively). More deaths during the first 24 h of admission were noted in patients with no endoscopy.

### 3.2. Mean Scores

We analyzed mean prognostic values in patients with variceal bleeding, non-variceal bleeding, and patients with no endoscopy performed to establish if patients with no endoscopy performed had more severe forms of UGIB (Table 12).

By comparing mean score values for patients with no endoscopy with those for variceal bleeding, mean modified Glasgow-Blatchford score was higher in patients with no endoscopy and pre-endoscopic Rockall and T-scores were higher in variceal bleeding, while other mean scores were similar. By comparing mean score values for patients with no endoscopy with those with non-variceal bleeding, most scores (Glasgow-Blatchford score, modified Glasgow-Blatchford score, pre-endoscopy Rockall and Baylor scores, Iino and MAP scores) were higher in patients with no endoscopy, while T-score mean value was lower; H3B2, Iino score, N-score, and modified N-score were similar. Considering the fact that higher values are associated with a higher risk for all scores with the exception of T-score, we can conclude that UGIB with no endoscopy was associated with a higher risk for mortality stratified by most pre-endoscopic scoring systems in comparison with non-variceal bleeding, while by comparing with variceal bleeding, the differences were not significant or were contradictory. The mean value for CCI in patients with no endoscopy was higher than in non-variceal bleeding and lower than in variceal bleeding, probably reflecting the presence of cirrhosis with liver failure in variceal bleeding.

### 3.3. Accuracy of Prognostic Scores

**Figure 1:**
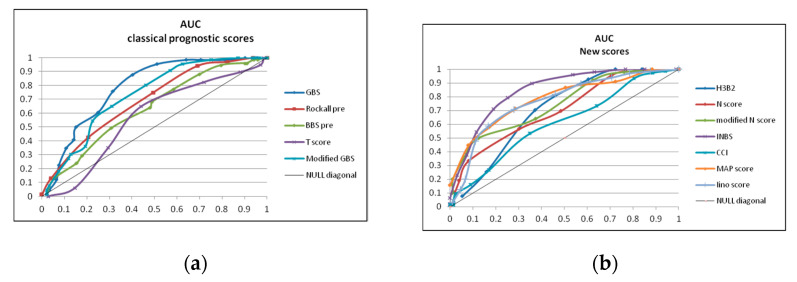
(**a**) Area under the curve for GBS, mGBS, AIM65, T-score and Baylor score (pre-endoscopic and after endoscopy); (**b**) New proposed scores: H3B2, Iino score, original and modified N-score International Bleeding score (INBS), MAP score and CCI.

Next, we analyzed the accuracy of prognostic scores for mortality prediction and we constructed AUC for the analyzed prognostic scores. The AUC, confidence interval, and *p*-value for the scores (Table 13) are illustrated in Figure 1.

A guideline proposed by Swets has assessed scoring systems by area operator under the curve (AUC) as non-informative (AUC = 0.5), less accurate (between 0.5 and 0.7), moderately accurate (between 0.7 and 0.9), highly accurate (between 0.9 and 1), and perfect (AUC = 1). In our study, the most accurate scores in patients with no endoscopy were the International Bleeding score (INBS) (AUC above 0.8), Glasgow Blatchford, MAP (ASH) score, Iino score, AIM65, modified Glasgow-Blatchford score, modified and original N-score, and H3B2, (all 8 scores being moderately accurate), while for Rockall, Baylor (pre-endoscopic), and T-scores, the AUC was between 0.563 and 0.7. The stratification of hematemesis in red (bloody) emesis and coffee-ground hematemesis did slightly improve the accuracy of the N-score.

### 3.4. CPT and MELD Score in Cirrhosis Patients

**Figure 2:**
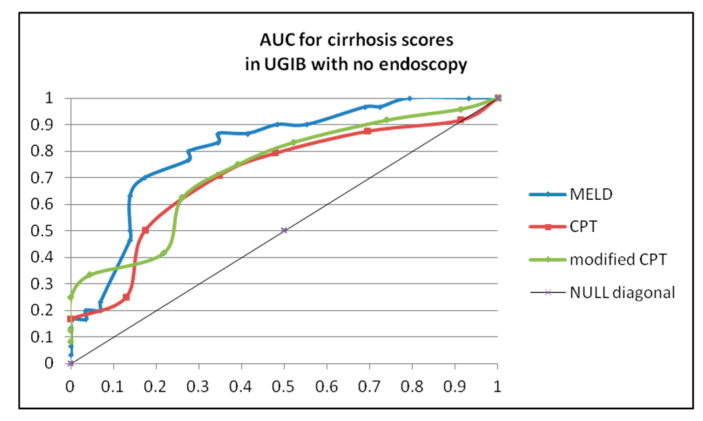
Area under curve for MELD, Child–Pugh–Turcotte and modified Child–Pugh–Turcotte (including creatinin level).

We assessed the accuracy of CPT and MELD scores for the evaluation of mortality risk in patients with no endoscopy and cirrhosis. AUC showed that MELD score had higher but moderate accuracy in patients with cirrhosis compared with CPT (0.811 versus 0.706). A modified variant of the Child–Pugh–Turcotte score, which included creatinine level, had a slightly improved accuracy (AUC 0.732)-Figure 2.

### 3.5. Reasons for no Endoscopy Performed: Pathology Examination

The main reasons for no endoscopy performed were denied consent in 35.4%, rapid death before endoscopy in 17.7%, cerebrovascular complications (deep coma, recent severe stroke, dementia, encephalitis, oligophrenia) in 14.1%, COVID-19 infection with respiratory failure in 8.6%, cardiopulmonary failure in 7.1%, lack of cooperation in 6.1%, and oro-pharyngeal or esophageal stenosis in 3%. COVID-19 infection with respiratory failure represents 19.8% of the reasons for not performing endoscopy in the pandemic period, where a conservative approach was recommended in the case of stable patients with no decreasing hemoglobin level. Postmortem pathologic examination was performed in 19 cases with no endoscopy performed and revealed esophageal ruptured varices in 7 cases (36%), hemorrhagic gastritis in 7 cases (36%), and hemorrhagic ulcer in 3 cases (15.8%); a slightly higher proportion of variceal bleeding in patients with no endoscopy was therefore suggested by the pathologic examination, although the difference was not statistically significant (36% versus 21.2%, OR1.83, 95%CI 0.72 to 4.66, *p* = 0.21).

## 4. Discussion

UGIB with unknown etiology includes a heterogenous group of patients in whom upper digestive endoscopy was not possible due to various reasons (denied consent, significant comorbidities especially cardio-respiratory failure, alcohol withdrawal, death shortly after admission, and other reasons). The mortality rate was much higher than in both variceal and non-variceal bleeding, was statistically similar in the pandemic period compared with the pre-pandemic period, and was higher in positive versus negative cases (62.1% versus 16.7%, *p* = 0.0007). Only one case of rebleeding was noted, which was resolved with conservative treatment. The hospitalization period was significantly shorter (with more than 2 days) compared with both variceal and non-variceal cases (*p* < 0.0001 and 0.0002, respectively).

Compared with non-variceal bleeding, age was similar but a lower male percentage was noted; comorbidities were similar to NVUGIB, with the exception of metastatic malignancies and a higher proportion of cirrhosis and a lower mean value of serum albumin were explained by a mixed NVUGIB and VUGIB population. Similar mean levels of hemoglobin but a lower blood requirement and a higher mean creatinine level were also observed. By analyzing the mean score value in variceal, non-variceal, and unknown etiology bleeding, we found that in patients with no endoscopy mean value of most scores reflect a higher risk than in NVUGIB (with the exception of N-score, H3B2 and Iino scores), while the differences from variceal bleeding were controversial and limited to few scores.

In our study, the most accurate scores were International Bleeding score (AUC above 0.8), followed by Glasgow Blatchford, MAP (ASH) score, Iino score, AIM65, modified Glasgow-Blatchford score, modified and original N-score, and H3B2 (all 8 scores being moderately accurate), while for Rockall, Baylor (pre-endoscopic), and T-scores, the AUC was between 0.563 and 0.7. Early risk stratification in UGIB was advocated by the 2010 International consensus guideline and also by the American College of Gastroenterology practice guidelines [2,24] by dividing patients with UGIB in high-risk and low-risk cases and establishing the level of care and the timing of endoscopy and discharge. Prognostic scores are the most used methods for risk stratification, although some studies showed that a significant number of physicians were not aware or did not use prognostic scores for UGIB in the emergency setting.

The accuracy of newly introduced scores in patients with no endoscopy was higher for the INBS and Iino score. Iino score and GBS shared some of the same components (systolic blood pressure, syncope, Hb level, and BUN), but Iino score introduced the presence of hematemesis, BUN/creatinine ratio and antiplatelet therapy as significant parameters. The stratification of hematemesis in red (bloody) emesis and coffee-ground hematemesis slightly improved the accuracy of N-score. The relatively poor performance of Rockall and Baylor bleeding scores in patients with no endoscopy performed was somewhat surprising.

There were many studies which assessed the accuracy of several scores regarding mortality prediction or therapeutic intervention, often with contradictory results. A systematic review published in 2016 which included 16 studies found that GBS score was more accurate for both intervention and 30-day mortality than Rockall score and AIM65 [30]; another study found that the accuracy of GBS for predicting mortality was poor [31], and in a study of NVUGIB, pre-endoscopic and full RS were superior to GBS in predicting in-hospital mortality (AUC 0.842 for pre-RS, 0.804 for full-RS and 0.622 for GBS) [25]. In a study of 237 patients with both variceal and non-variceal UGIB, RS was superior to GBS [26]. A study which analyzed in-hospital mortality found that AIM65 was better than both GBS and full Rockall score in predicting mortality (AUC 0.955), regardless of variceal or non-variceal bleeding [24], while another study found that the accuracy of AIM65 was better than that of GBS for predicting 30-day mortality (AUC 0.706 versus 0.542) [32]. A retrospective study of six scoring systems (AIM65, GBS, MAP, T-score, ABC and pre-endoscopic Rockall score) found that the accuracy for mortality was highest for ABC score followed by MAP (similar to our study), while for predicting intervention, pre-endoscopic Rockall score was the only useful score. In cancer patients with acute UGIB, AIM65 was better than GBS or Rockall score in predicting mortality (AUC 0.84) [33]. Another study of 5 scores (pre-RS, full-RS, GBS, modified GBS and AIM65) and 7 outcomes found that the need for transfusion and for surgery was predicted by GBS and modified GBS, 30-day mortality by both pre-endoscopic and full Rockall score but also by AIM65, and the need for endoscopic therapy and admission in ICU was predicted by all 5 scores; none of the scores predicted rebleeding risk [3]. A multicenter study which assessed the accuracy of 5 scores (GBS, Pre-RS, full-RS, AIM65, and PNED) for both intervention and mortality found that GBS was superior in predicting intervention or death [34]. Another study which analyzed the performance of 3 scores (GBS, full Rockall score and CS) for the prediction of in-hospital mortality found that all scores had an AUC > 0.9 but CS was superior to RS and GBS [4].

Some studies have even proposed cut-off values for main scores for several outcomes (2.5 for AIM65, 11.5 for GBS, 1.5 for pre-endoscopic Rockall score [1], (3 for AIM65, 14 GBS, 5 CCI) [35]. In a study, the optimal cut-off for mortality for pre-endoscopic Rockall score was 9, and the prediction of 30-day mortality was best realized by the pre-endoscopic Rockall score, while the prediction for the need for intervention was best obtained by using Glasgow-Blatchford original or modified scores [2]. A Rockall score < 2 was associated with a low risk for mortality [36], while pre-endoscopic Rockall score > 4 was associated with a high risk for 30-day mortality, which suggests a more aggressive approach in these patients [2]. In our study, cut-off values for mortality were 7 for INBS, 4 for MAP, Iino, pre-endoscopic Rockall, N-score and H3B2, 11 for GBS, 10 for T-score, 9 for modified GBS, 2 for AIM65, and 3 for modified N-score.

Charlson comorbidity index represents a method for estimating the concomitant diseases score and estimating mortality risk by weighing associated diseases, but the importance in UGIB prognosis is currently unclear; the accuracy seems low in the context of UGIB, especially in 30-day mortality [35]. In our study, CCI had a poor accuracy in UGIB (AUC 0.609).

There are some limitations in the study. The retrospective design is associated with incomplete data for some of the cases. Excluding cases with only some scores available limited statistical evaluation to 122 patients by not including the modified N-score and to 101 patients with the inclusion of the proposed modified N-score. Additionally, the number of analyzed scores was large, some of the scores are not very familiar to many doctors, and several scores are newly introduced with only a few studies available in the literature, so our study may seem too exhaustive. Still, UGIB patients without endoscopy are an understudied category of patients, and also, somewhat surprisingly, most newly introduced scores performed better than some older scores (such as the T and Baylor scores). Considering these results, we believe that a comprehensive study of the most available prognostic scores in UGIB without endoscopy is critical to evaluate the prognostic significance in this particular group of patients.

## 5. Conclusions

UGIB with no endoscopy performed had a higher mortality rate compared to both variceal and non-variceal bleeding, and had mostly higher prognostic scores compared with non-variceal bleeding, while by compared with variceal bleeding, the differences regarding prognostic scores were not significant or were contradictory. In our study, the most accurate scores were INBS (AUC above 0.8), Glasgow Blatchford, MAP (ASH) score and Iino score (AUC between 0.7 and 0.8), while for Rockall score, Baylor score, and T-score, AUC was between 0.563 and 0.7. In patients with no endoscopy and cirrhosis, MELD score had a higher but moderate accuracy compared with CPT. In patients with no endoscopy, the mortality rate was higher in the pandemic period but with no statistical significance and was higher in positive cases (62.1% versus 16.7%, OR = 8.1818, 95% CI 2.4192 to 27.6707, *p* = 0.0007).

## Figures and Tables

**Table 1:** Rockall score [3].

	0	1	2	3
Age	<60	60–79	≥80	-
Shock	*p* < 100 sBP ≥ 100	*p* ≥ 100 ≥100	sBP < 100	-
Comorbidities	NO major	-	Cardiac failure, coronary ischemia	Renal/liver failure Disseminated malignancy
Diagnosis	MW No lesion No stigmata	Other exc. malignancy	Malignancy	
Bleeding stigmata	No/ dark spot	-	Blood, adherent clot Visible/spurting vessel	-

*p* = pulse, sBP = systolic Blood Pressure, MW = Mallory-Weiss syndrome.

**Table 2:** Glasgow-Blatchford score [7].

Urea (mg/dL)	39–47	2
	48–60	3
	60–149	4
	≥150	6
Hb (g/dL)	Men 12–12.99	1
	Men ≥ 10	3
	Woman ≥ 10	1
	Both sexes < 10	6
sBP (mm Hg)	100–109	1
	90–99	2
	<90	3
Pulse (>100/min)		1
Melena		1
Syncope		2
Liver disease		2
Cardiac failure		2

sBP = systolic blood pressure.

**Table 3:** Baylor score [28].

	1	2	3	4	5
Age	30–49	50–59	60–69		≥70
Number of diseases	1–2			3–4	≥5
Severity of diseases				Chronic	Acute
Endoscopy score					
Site of bleeding				Posterior DU	
Bleeding stigmata	IIb		IIa		I

DU = duodenal ulcer.

**Table 4:** T-score.

	1	2	3
Pulse	>110	90–110	<90
Systolic blood pressure	<90	90–110	>110
Hemoglobin	≤8 g%	9–10	>10
General condition	Poor	Intermediate	Good

**Table 5:** AIM65 score [7].

	Score
Age > 65 year	1
Systolic blood pressure < 90	1
Altered mental status	1
Alb < 3 g%	1
INR > 1.5	1

**Table 6:** N-score and proposed m-N-score [8].

	N-Score	m-N-Score
Syncope	3	3
Hematemesis	2	
Red blood hematemesis		2
Coffee-ground hematemesis		1
BUN ≥ 22.4	1	1
BUN/creatinine ≥ 30	1	1

BUN = Blood Urea Nitrogen; m-N-score = modified N-score.

**Table 7:** H3B2 score [7].

	Points
Hematemesis	1
Heart rate (times/minute) ≥ 100	1
Blood pressure (systolic) ≤ 100 mm Hg	1
Hb ≤ 10 g/dL	1
BUN ≥ 22.4	2

BUN = Blood Urea Nitrogen.

**Table 8:** MAP score modified after [7].

	Points
Disturbance of consciousness	1
ASA score > 2	1
Heart rate (times/minute) > 100	1
Blood pressure (systolic) < 90 mm Hg	2
Hb < 10 g/dL	2
Albumin < 2.5 g/dL	2

ASA score = American Society of Anesthesiology score.

**Table 9:** Iino score [7].

	Points
Blood pressure (systolic) < 100 mm Hg	2
Syncope	2
Hematemesis	3
Hb < 10 g/dL	1
BUN ≥ 22.4	2
eGFR (ml/min/1.73 mm) ≥ 60	−2
Oral antiplatelet drug	−2

BUN = Blood Urea Nitrogen; eGFR = estimated Glomerular Filtration Rate. CKD-EPI Creatinine Equation (2021) was used [29].

**Table 10:** INBS (ABC) score [17].

	Points
Age	
60–74 years	1
≥75 years	2
Comorbidity	
Altered mental status	2
Liver cirrhosis	2
Disseminated malignancy	2
ASA score	
3	1
≥4	3
Blood tests	1
Urea > 10 mmol/L	1
Albumin < 30 g/L	2
Creatinine	
100–150 μmol/L	1
>150 μmol/L	2

ASA score = American Society of Anesthesiology score.

**Table 11:** Main characteristics of patients with unknown etiology (no endoscopy) compared with VUGIB and NVUGIB.

	Variceal (N = 520)	Non-Variceal (N = 1628)	Unknown (N = 198)
**Age** (years, min–max)	58.7 (21–85)	63.9 (17–95)	65 (26–99)
<60	47.3	35.1	33.8
60–79	51.2	51.1	53.0
>80	1.5	13.8	13.1
**M** (%)	342 (65.8)	1101 (67.6)	119 (60.1)
**Comorbidities**			
Cirrhosis (%)	503 (96.7)	179 (11)	71 (35.9)
Renal disease (%)	8 (1.5)	125 (7.7)	14 (7.1)
Cardiac disease (%)	17 (3.3)	242 (14.9)	25 (12.6)
Metastatic malignancy (%)	3 (0.6)	6 (0.4)	9 (4.6)
**Medication**			
Antiplatelet agents (%)	3 (0.6)	106 (6.5)	6 (3)
Anticoagulants (%)	9 (1.7)	217 (13.3)	8 (4)
NSAID (%)	16 (3.1)	250 (15.4)	8 (4)
**Alcohol abuse**	365 (70.2)	519 (31.9)	72 (36.4)
**Mean onset-adm. time** (h)	24.4	46.7	37.1
**Hematemesis** (%)	78.7	45.8	50
**Melena** (%)	89.6	86.9	81.3
**Hematochezia** (%)	5	2.6	10.1
**Laboratory analysis**			
Hb	8.28	8.77	8.39
Urea	65.1	82.6	90.7
Creatinine	0.96	1.24	1.56
INR	1.74	1.58	1.77
Albumin	2.70	3.27	2.82
**Mean adm. to endo time** (h)	18.5	18.4	NA
**Admission to endoscopy** (%)			
<12 h	65.5	58	NA
12–24 h	20	25.9	NA
>24 h	14.5	16.1	NA
**Blood transfusions%**	55.2	45.9	34.8
**Rebleeding rate (%)**	38 (7.3)	68 (4.2)	1 (0.5)
**Mean hospital stay (days)**	7.95	7.39	5.47
**In-hospital mortality**	114 (21.9)	121 (7.4)	75 (37.9)
Death during first 24 h adm.	17 (14.9)	13 (10.7)	17 (22.7)

Adm. = admission.

**Table 12:** Mean prognostic scores in patients with no endoscopy performed.

	No Endoscopy	VUGIB	*p*-Value	NVUGIB	*p*-Value
**Scores (Mean ± SD)**					
***Classic scores***					
Glasgow Blatchford	10.60 ± 4.11	10.71 ± 3.42	0.7574	9.28 ± 3.69	*<0.0001*
Glasgow Blatchford modified	8.44 ± 3.72	7.66 ± 3.25	*0.0119*	7.62 ± 3.34	*0.0038*
Rockall pre-endoscopy	3.62 ± 1.51	**4.16 ± 0.96**	*<0.0001*	2.71 ± 1.69	*<0.0001*
Baylor pre-endoscopy	9.07 ± 3.37	9.12 ± 2.25	0.8193	8.09 ± 4.16	*0.0014*
AIM65	1.76 ± 1.19	1.68 ± 0.99	0.4464	1.12 ± 0.99	*<0.0001*
T-score	9.62 ± 1.59	9.98 ± 1.43	*0.0070*	9.95 ± 1.51	*0.0082*
***New scores***					
N-score	3.01 ± 1.86	3.15 ± 1.66	0.3761	2.90 ± 1.85	0.4400
Adjusted N-score	2.64 ± 1.88	2.82 ± 1.72	0.3046	2.54 ± 1.78	0.5361
H3B2	3.25 ± 1.61	3.43 ± 1.47	0.2009	3.12 ± 1.49	0.2752
Iino score	3.22 ± 2.96	3.22 ± 2.31	0.9930	2.50 ± 2.53	*0.0009*
MAP (ASH) score	3.34 ± 2.01	3.54 ± 1.65	0.2565	2.38 ± 1.60	*<0.0001*
INBS score					
***Charlson comorbidity index (CCI)***	4.28 ± 2.01	**4.76 ± 1.45**	*0.0004*	3.37 ± 2.24	*<0.0001*

SD = standard deviation; VUGIB = Variceal upper gastrointestinal bleeding; NVUGIB = non-variceal upper gastrointestinal bleeding. Statistically significant *p*-values are marked with italicized fonts. Higher risk values of mean scores are marked with bold fonts; lower risk values are marked with underlined fonts.

**Table 13:** Scores for patients with no endoscopy.

	AUC	95% CI	Significance
Glasgow Blatchford	*0.783*	0.712–0.854	0.000
Glasgow Blatchford (adjusted)	*0.730*	0.653–0.807	0.000
Rockall pre-endoscopy	0.686	0.606–0.766	0.000
Baylor pre-endoscopy	0.632	0.554–0.710	0.002
AIM65	*0.745*	0.660–0.829	0.000
T-score	0.563	0.471–0.656	0.168
N-score	*0.701*	0.622–0.780	0.000
mN-score	*0.745*	0.659–0.831	0.000
H3B2	*0.701*	0.621–0.780	0.000
Iino score	*0.766*	0.690–0.842	0.000
MAP (ASH) score	*0.780*	0.696–0.865	0.000
INBS	**0.844**	0.781–0.906	0.000
CCI	0.609	0.529–0.688	0.000
CPT score	0.706	0.557–0.857	0.016
Modified CPT	0.732	0.587–0.877	0.003
MELD score	**0.811**	0.699–0.923	0.000

AUC values above 0.8 are marked with Bold fonts, and values above 0.7 (moderately accurate) are marked with italicized fonts. CPT = Child–Pugh–Turcotte, CCI = Charlson comorbidity index.

## Data Availability

The datasets generated during and/or analyzed during the current study are available from the corresponding author on reasonable request.

## Abbreviations

BBS	Baylor bleeding score
CCI	Charlson comorbidity index
CPT	Child–Pugh Turcotte score
CSMCPI	Cedars–Sinai Medical Center Predictive Index
GBS	Glasgow-Blatchford score
ICU	intensive care unit
INBS	International Bleeding Score
mGBS	modified Glasgow-Blatchford score
m-N-score	modified N-score
NVUGIB	non-variceal upper gastrointestinal bleeding
PNED	Progetto Nazionale Emmoragia Digestive
RS	Rockall score
TSC	T-score
UGIB	upper gastrointestinal bleeding
VUGIB	variceal upper gastrointestinal bleeding
